# Anal gland adenocarcinoma in situ with pagetoid spread: a case report

**DOI:** 10.1186/s40792-018-0469-5

**Published:** 2018-06-25

**Authors:** Kohei Ishioka, Fumikazu Koyama, Hiroyuki Kuge, Takashi Inoue, Shinsaku Obara, Takayuki Nakamoto, Yoshiyuki Sasaki, Yasuyuki Nakamura, Maiko Takeda, Chiho Ohbayashi, Masamitsu Kuwahara, Masayuki Sho

**Affiliations:** 1 Department of Surgery, Nara Medical University, 840 Shijo-cho, Kashihara, Nara 634-8522 Japan; 2 Department of Endoscopy, Nara Medical University Hospital, 840 Shijo-cho, Kashihara, Nara 634-8522 Japan; 3 Department of Diagnostic Pathology, Nara Medical University, 840 Shijo-cho, Kashihara, Nara 634-8522 Japan; 4 Department of Plastic Surgery, Nara Medical University, 840 Shijo-cho, Kashihara, Nara 634-8522 Japan

**Keywords:** Anal gland adenocarcinoma, Adenocarcinoma in situ, Perianal Paget’s disease

## Abstract

**Background:**

Anal gland carcinoma with perianal Paget’s disease is rare, and anal gland carcinoma in situ is extremely rare. No cases of anal gland carcinoma in situ with pagetoid spread have been previously reported.

**Case presentation:**

Physical examination in a 75-year-old woman revealed an erythematous, inflamed, perianal skin lesion. Neither colposcopy, cystoscopy, colonoscopy, computed tomography, nor magnetic resonance imaging showed evidence of malignant genitourinary or gastrointestinal lesions. Histopathological examination of a biopsy specimen showed many Paget’s cells in the perianal skin lesion and no malignant cells in the rectal or vaginal mucosa. Therefore, primary extramammary Paget’s disease of the anogenital region was suspected, and we performed anus-preserving wide local excision. However, immunohistochemistry revealed a diagnosis of secondary extramammary Paget’s disease due to adenocarcinoma arising from the anal gland. We therefore proceeded with a radical operation. Histopathological examination showed no residual cancer cells. The final diagnosis was anal gland adenocarcinoma in situ with pagetoid spread in the perianal skin.

**Conclusions:**

This is the first case report of anal gland adenocarcinoma in situ with pagetoid spread. We recommend immunohistochemical analysis of biopsy and locally resected specimens to obtain an accurate diagnosis and determine the appropriate treatment when there is no visible tumor.

## Background

Perianal Paget’s disease (PPD) is rare. There are two types of PPD: primary and secondary. When an underlying adenocarcinoma is present, PPD usually represents intraepidermal extension of an invasive carcinoma from an adjacent internal organ and is generally regarded as secondary [1].

There are some reports of pagetoid spread of adenocarcinoma in situ of the cervix [2] and pagetoid changes in urothelial carcinoma in situ [3, 4]. However, there are no reports of anal gland adenocarcinoma in situ with pagetoid spread.

Herein, we report the first documented case of anal gland carcinoma in situ with pagetoid spread.

## Case presentation

A 75-year-old woman was admitted to our hospital complaining of itching around her anus. She had a history of sigmoidectomy for diverticulitis 6 years prior and a past history of Sjögren’s syndrome. She had noted a reddish skin lesion around her anus over the past several years. She reported no change in bowel habits, no gastrointestinal symptoms, no weight loss, and no family history of malignancy.

**Fig. 1:**
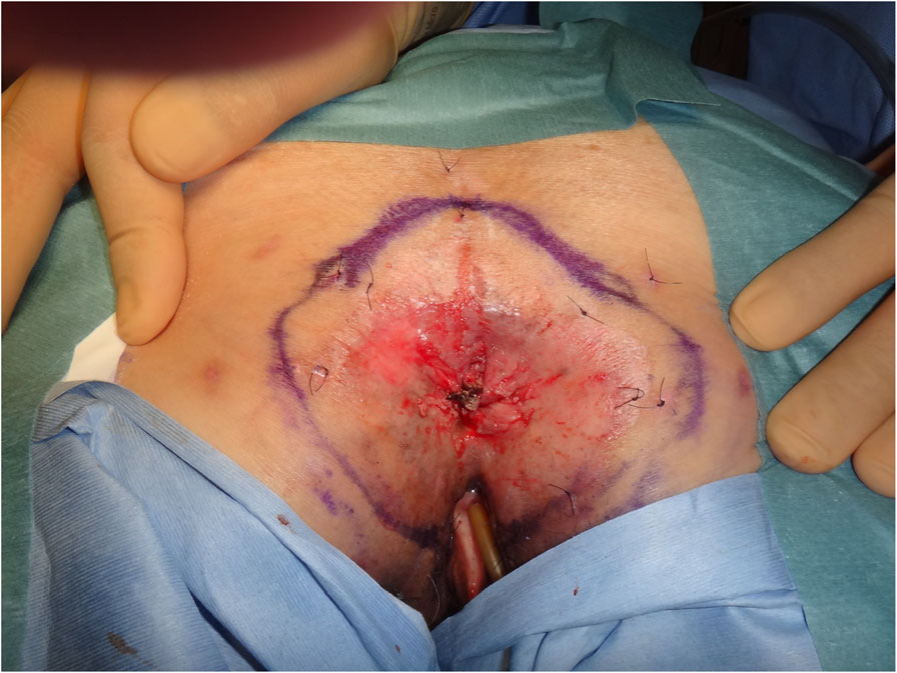
Macroscopic view of the perianal skin. A 60 × 75-mm pagetoid spreading lesion is visible around the anus

**Fig. 2:**
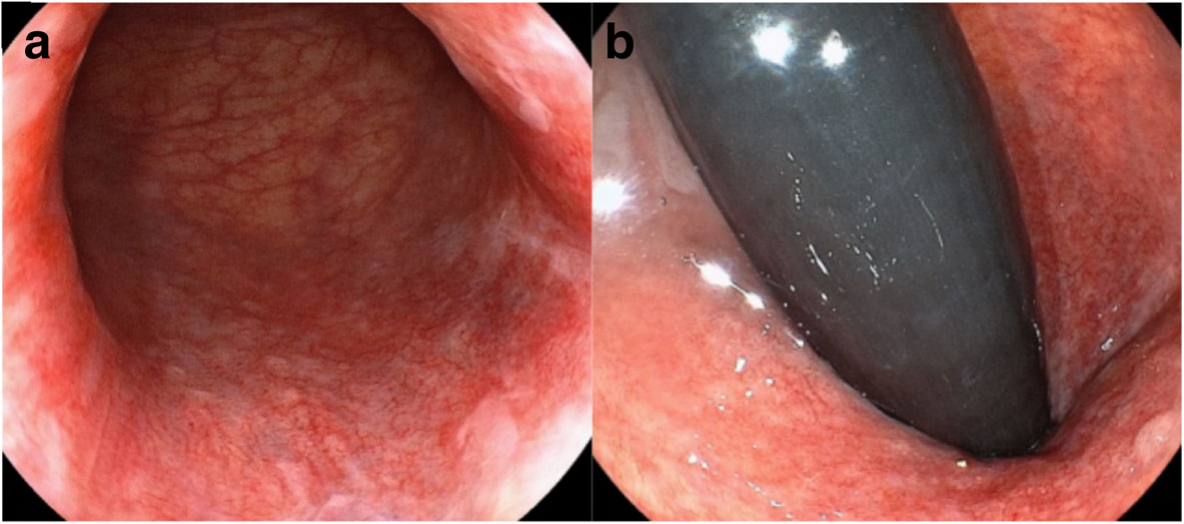
Colonoscopy. There is no visible lesion in the rectum or anal canal. **a** Ordinary observation. **b** Reversal observation

**Fig. 3:**
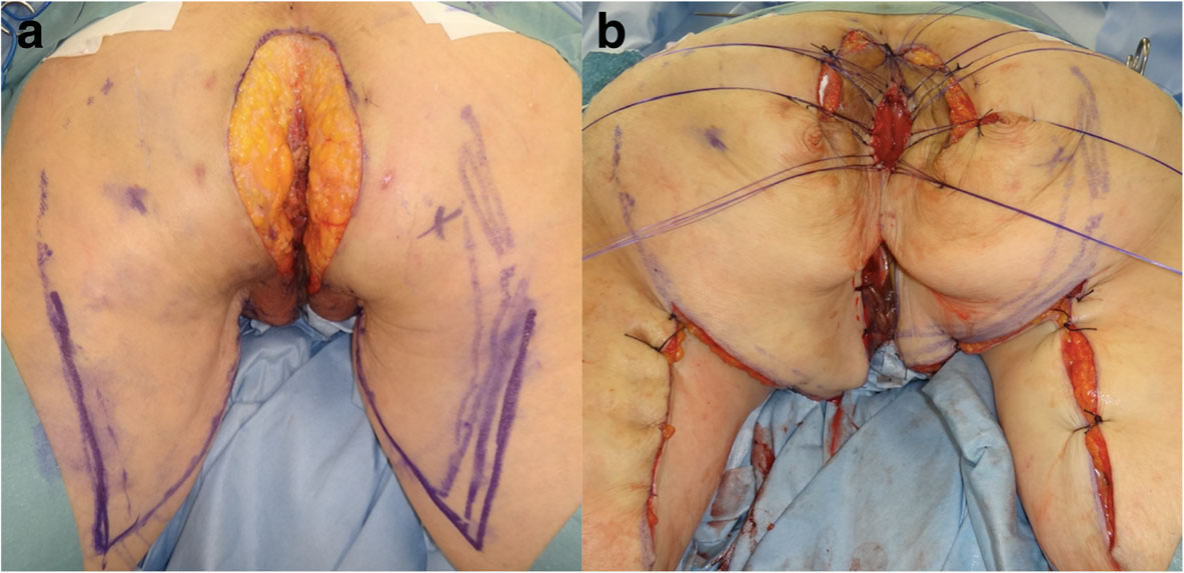
Surgical findings. **a** Anal-preserving wide local excision deep to the subcutaneous fat. **b** Reconstruction using a bilobed gluteal fold flap

Physical examination revealed an erythematous, inflamed skin lesion in the perianal region (Fig. 1), but a normal vagina and rectum. Neither colposcopy, cystoscopy, nor colonoscopy showed evidence of a visible lesion or any abnormality of the cervix, bladder, or rectum (Fig. 2a, b). Computed tomography and magnetic resonance imaging showed no evidence of malignancy in the genitourinary or gastrointestinal tracts. Histopathological examination of biopsy specimens showed many Paget’s cells within intraepithelial lesions of the perianal skin but no malignant cells in the rectal or vaginal mucosa. Therefore, primary extramammary Paget’s disease (EMPD) of the anogenital region was suspected. We performed anal-preserving wide local excision deep to the subcutaneous fat with 1-cm negative margin from the positive sites confirmed by frozen section examination and mucosal resection of the anal canal that was extended 1 cm proximal to the dentate line of the anal canal. Reconstruction was performed using a bilobed gluteal fold flap (Fig. 3a, b).

**Fig. 4:**
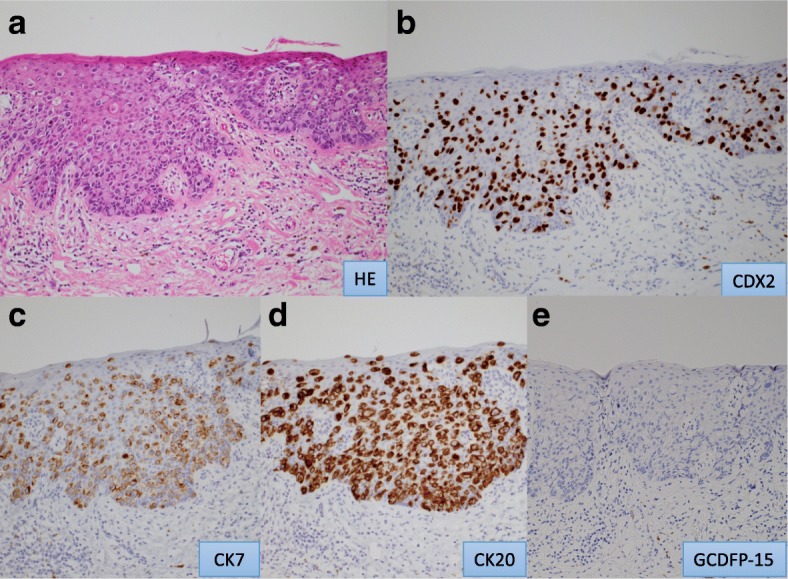
Histological findings of perianal Paget’s disease. **a** Paget’s cells with clear cytoplasm and large pleomorphic nuclei (hematoxylin-eosin; magnification × 20). **b**–**d** Positive immunohistochemical staining for cytokeratin (CK) 7 (**b**), CK20 (**c**), and caudal-related homeobox gene nuclear transcription factor (CDX) 2 (**d**), and negative for gross cystic disease fluid protein (GCDFP) 15 (**e**) (magnification × 20)

**Fig. 5:**
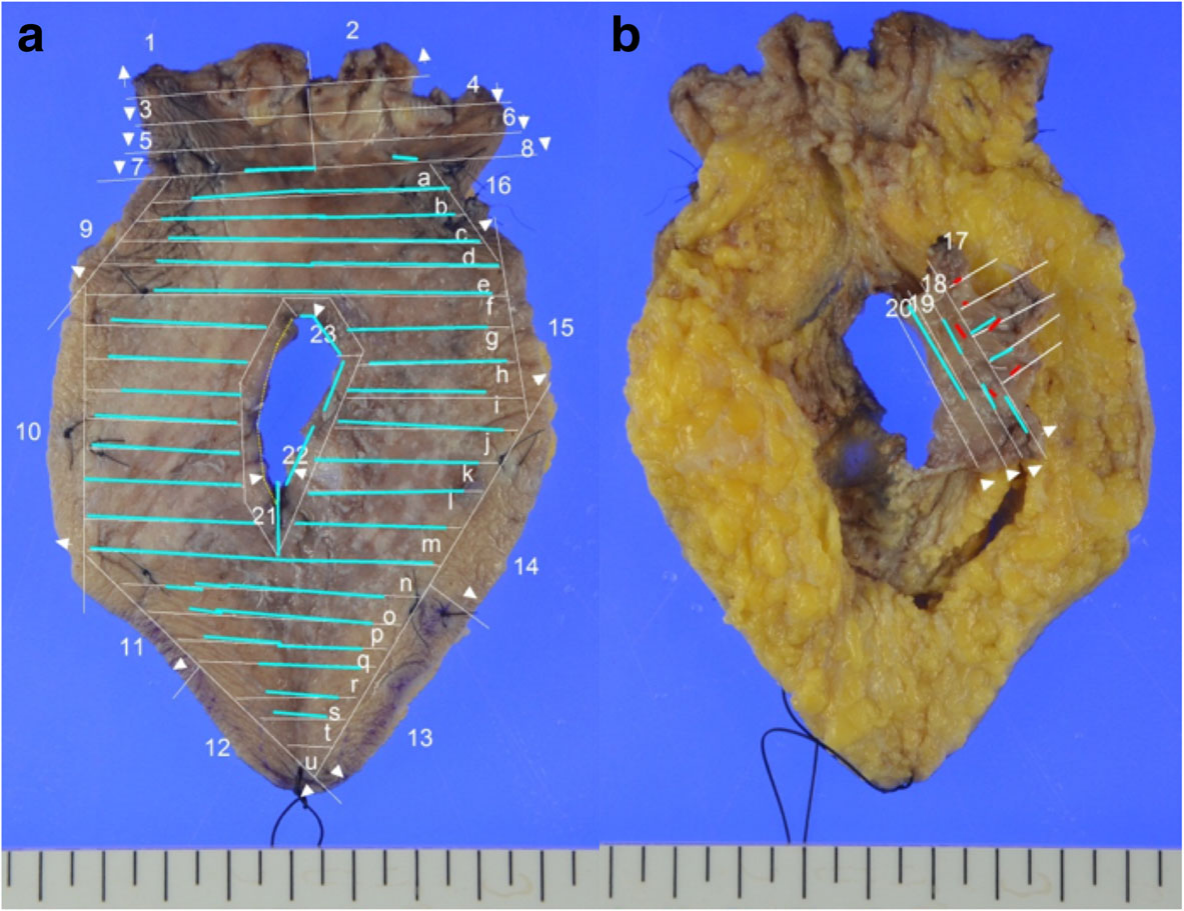
Macroscopic images of locally resected specimen. Blue lines indicated pagetoid spread, and red lines indicated anal gland adenocarcinoma in situ. **a** Skin side. **b** Back side

**Fig. 6:**
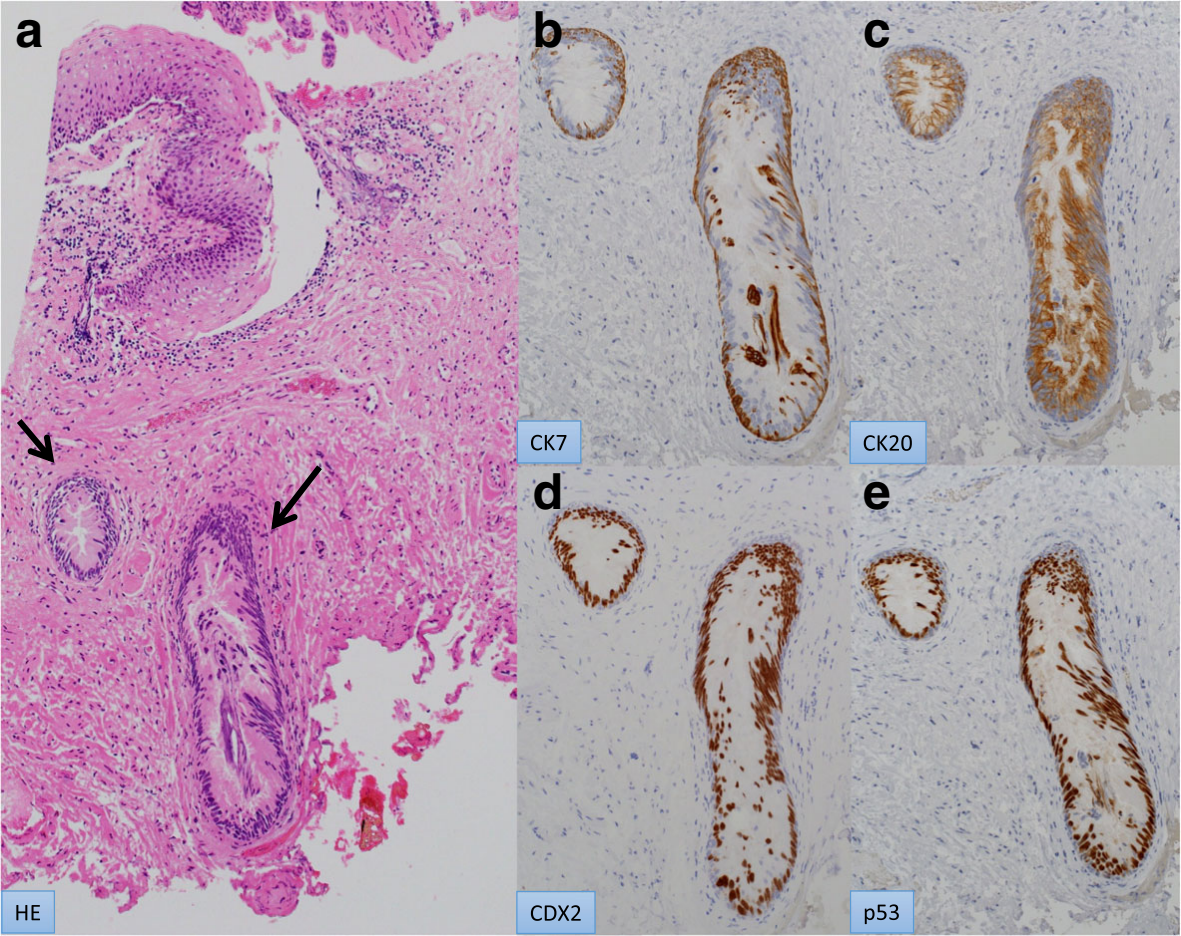
Histological findings of anal gland carcinoma. **a** In situ adenocarcinoma of the anal glands (arrow) (hematoxylin-eosin; magnification × 10). **b**–**e** Positive immunohistochemical staining for CK7 (**b**), CK20 (**c**), CDX2 (**d**), and p53 (**e**) (magnification × 20)

Histopathological examination of the resected specimen showed Paget’s cells, characterized by clear cytoplasm and large pleomorphic nuclei, within the epidermis. Immunohistochemical analysis revealed that the Paget’s cells were positive for cytokeratin (CK) 7, CK20, and caudal-related homeobox gene nuclear transcription factor (CDX) 2 and negative for gross cystic disease fluid protein (GCDFP) 15 (Fig. 4a–e). These data suggested that her perianal skin lesion was secondary EMPD arising from the rectum, although there was no lesion found in the rectum. Additional histopathological examination of the resected specimen revealed well-differentiated adenocarcinoma in an anal gland, continuous with the Paget’s cells in the anoderm, but limited in the basement membrane without a desmoplastic change (Figs. 5a, b and 6a–e). Immunohistochemical staining of the resected specimen revealed secondary EMPD due to adenocarcinoma arising from the anal gland.

Because of the possibility of residual adenocarcinoma in the anal gland, possibly extending to the sphincter, we performed a radical laparoscopy-assisted abdominoperineal resection. Any reconstructive plastic surgery was not needed. Histopathological examination revealed no residual cancer cells in the resected specimen and no lymph node metastasis. The final diagnosis was anal gland adenocarcinoma in situ with pagetoid spread in the perianal skin.

## Discussion

PPD was defined as intraepithelial adenocarcinoma of the perianal skin and is classified into two subgroups: primary and secondary [5]. Primary PPD originates from the epidermis or skin appendages, including the eccrine glands, apocrine glands, ectopic mammary-like glands, and epidermal pluripotent stem cells. Secondary PPD, also known as pagetoid phenomenon, is a metastatic tumor derived from underlying carcinoma cells [1]. Immunohistochemical staining for GCDFP15, CDX2, CK7, and CK20 are especially useful in differentiating the type and the origins of EMPD. GCDFP15 is considered as apocrine epithelium-specific tissue marker [1], and negative in secondary EMPD [6]. The expression of CDX2 is a sensitive and specific marker for the secondary type, arising from anorectal or colonic adenocarcinoma [7], but CDX2 fails to distinguish primary disease from EMPD secondary to urothelial or prostatic malignancy [8]. Many anal gland adenocarcinomas stained for CK7 but did not stain for CK20, similar to the normal anal gland [9–11]. However, several cases of secondary type arising from anal gland carcinoma showed CK7 positive and CK20 positive [12–14].

Approximately 50% of patients with anal-margin Paget’s disease harbor a colorectal neoplasm mandating full colonoscopy for complete evaluation [15]. In our patient, there was no visible gastrointestinal, genital, or urinary tumor, and we initially suspected primary PPD. However, immunohistochemical analysis (CK7 +, CK20 +, GCDFP15 −, CDX2 +) suggested secondary EMPD. This discrepancy led us to perform additional histopathological studies on the resected specimen, which revealed the original site of the Paget’s disease as an in situ, well-differentiated adenocarcinoma in an anal gland.

Anal crypt glands, which lie at the level of the dentate line, can extend into the longitudinal layer after penetrating the internal sphincter [16, 17]. Thus, combined resection of the anal sphincter at the site of the malignancy is necessary for curative resection of secondary EMPD arising from an anal gland, even if the lesion is carcinoma in situ.

## Conclusions

This case report indicates that the symptomatic pagetoid changes in the perianal skin without a visible tumor in the rectum should raise the suspicion of pagetoid spread from anal gland adenocarcinoma in situ. We recommend immunohistochemical analysis of biopsy specimen to elucidate the differential diagnosis of primary vs secondary Paget’s disease, and locally resected specimen to identify the origin of secondary EMPD and determine the appropriate treatment.

## Abbreviations

CDX: Caudal-related homeobox gene nuclear transcription factor
CK: Cytokeratin
EMPD: Extramammary Paget’s disease
GCDFP: Gross cystic disease fluid protein
PPD: Perianal Paget’s disease
